# Relationship between Fractal Dimension and Properties of Engineered Cementitious Composites with Different Aggregates

**DOI:** 10.3390/ma15217666

**Published:** 2022-10-31

**Authors:** Duotian Xia, Ruilin Chen, Duo Zhang, Jianjun Cheng

**Affiliations:** 1 College of Water Conservancy & Architectural Engineering, Shihezi University, Shihezi 832003, China; 2 Xinjiang Production & Construction Groups Engineering Laboratory for Seismic and Energy-Saving Building in High Earthquake Intensity and Cold Zone, Shihezi 832003, China; 3 Jinchuan Nickel Cobalt Research & Design Institute Co., Ltd., Jinchang 737100, China

**Keywords:** engineered cementitious composites, polyethylene fiber, desert sand, uniaxial tensile, fractal dimension

## Abstract

In this study, the effects of different fine aggregates on the properties of polyethylene fiber engineered cementitious composite (PE-ECC) were systematically investigated. The PE-ECCs were prepared with four fine aggregates, respectively. Furthermore, their flowability, compressive strength, and uniaxial tensile properties were studied experimentally and comparatively analyzed by microscopic techniques including X-ray diffraction (XRD), scanning electron microscope (SEM), energy-dispersive spectroscopy (EDS), and mercury intrusion porosimetry (MIP). The results showed that all the different types of fine aggregates exhibited little effect on the flowability of PE-ECC, but a greater effect on the compressive strength, uniaxial tensile strength, and ultimate tensile strain. PE-ECC prepared from untreated desert sand showed the best comprehensive performance, with compressive strength, uniaxial tensile strength, and ultimate tensile strain of 47.92 MPa, 6.26 MPa, and 3.638%, respectively. Moreover, it was found that the ultra-fine particles in the desert sand promoted the hydration reaction of cement and produced more C–S–H gels. The pore structures of ECC prepared with different aggregates exhibited obvious fractal characteristics, and the fractal dimension ranged from 2.8 to 2.9. The fractal dimension showed a strong correlation with parameters including ultimate tensile strain and pore structure, and the larger the fractal dimension, the smaller the ultimate tensile strain, porosity, and average pore size of ECC.

## 1. Introduction

Engineered cementitious composites (ECCs) with high ductility and high toughness as well as the advantage of achieving multiple cracking loads [1] have attracted significant research attention on the global scale as an efficient replacement for concrete and potential candidate with frequent applications in practical engineering structures. However, the concrete fine aggregate used for the preparation of ECC-standard sand, and even its substitute river sand exhibit serious imbalance between supply and demand and the cost is also gradually increasing, thus the world is facing potentially disastrous shortage of standard sand [2]. Nonetheless, at the same time, desert sand resources are abundant and the cheapest material in desert area, and the fine grain size of desert sand is also more suitable for the requirements of ECC materials.

Current studies on desert sand-based ECC mainly focus on its macroscopic properties as well as its microscopic morphology [3,4,5,6,7,8,9,10,11,12,13,14,15,16], and a few of them compared the performance differences between ECC prepared from desert sand and ECC prepared from other sands [11,12,13,14,15,16]. Comparative analysis reported in a literature study [11] indicated that desert sand-based polyvinyl alcohol engineered cementitious composites (PVA-ECC) exhibited superior uniaxial tensile properties than river sand PVA-ECC, and desert sand could improve the interfacial properties of PVA and matrix and enhance the fiber pull-out slip. Moreover, another study showed that the flexural properties of river sand-based ECC panels and desert sand-based ECC panels were relatively close [12] and the compressive ductility and toughness as well as the bending properties of desert sand-based ECC far exceeded those of river sand-based ECC [13]. However, Yang et al. [14] found that the hydration of desert sand-based ECC was slower than that of ordinary sand-based ECC, which was analyzed because of the higher C–H production that hindered the cement hydration rate. Li et al. [15] prepared ECC by completely replacing microquartz sand with desert sand and found that desert sand-based ECCs were comparable to microquartz sand-based ECCs in terms of mechanical properties such as tensile and compressive resistance. However, they were less ductile as well as less capable of bending densification. Sheng et al. [16] found that the tensile resistance of ECC prepared from desert sand was better than that prepared from standard sand; however, worse than that prepared from river sand, which might be caused by the existence of high number of large pores in desert sand-based ECC. The above-mentioned studies proved that it is feasible to use desert sand to prepare ECC; nonetheless, different scholars have reached different conclusions. We also found that compared with the commonly used fine aggregates such as river sand, desert sand exhibits a small grain size offset and poor grading, which results in a difference in the pore structure of the ECC prepared from desert sand and ordinary sand, which in turn produces a huge difference in the mechanical properties of ECC.

Furthermore, many studies on the pore structure of concrete and ordinary sand ECC have been reported till date. The characterization parameters of pore structure in concrete include pore diameter distribution, pore surface area, porosity, etc. However, it is not accurate to express the pore structure by using only single parameters. Notably, fractal dimension shows advantages in characterizing the discontinuity and irregularity of the pore structure [17,18,19], which can effectively quantify and compare the complexity of the material pore structure. Zeng et al. [17] investigated the fractal dimension of cement paste and mortar with/without ground granulated blast-furnace slag (GGBS) and obtained by different maintenance methods. The results confirmed that both maintenance methods and GGBS affected the fractal dimension; moreover, it was also demonstrated that the fractal dimension was related to the material water–cement ratio, curing age, and fly ash content [20]. Wang et al. [21], on the one hand, investigated the pore structure and fractal dimension of concrete prepared with fly ash and silica fume, and revealed the relationship between the porosity of concrete and the fractal dimension. The results showed that the fractal dimension affected the porosity of concrete. On the other hand, the relationship between pore structure parameters and fractal dimension was investigated, and the results showed that the pore fractal dimension exhibited a more significant effect on the porosity and the most probable pore size [22]. Zhang et al. [23] explored the relationship between the fractal dimension of high-strength cementitious materials and their pore structure parameters and compressive strength. The results revealed that the pore structure of high-strength cementitious materials exhibited obvious fractal characteristics, and the correlation between the fractal dimension and the compressive strength and porosity of the materials was found to be poor. The larger the fractal dimension, the larger the pore diameter and the larger the pore surface area of high-strength cementitious materials, and the fewer the pores smaller than 20 nm and larger than 100 nm. Clearly, the fractal dimension shows a significant correlation with the material properties and pore structures, and different aggregates cause large differences in the pore structure of the material. At present, information regarding the relationship between the fractal dimension of desert sand-based ECC materials and their pore characteristics and properties still lacks, as well as the similarities and differences between fractal dimensions of desert sand-based ECC and ordinary sand-based ECC materials have never been investigated till date.

In summary, the use of local resource desert sand to replace fine aggregate in traditional ECC can not only aid in reasonable utilization of natural resources and cost saving, but also be conducive to the regionalized development and application of ECC. Moreover, comparative analysis of the differences in pore structure between ECCs prepared from some different fine aggregates including desert sand and investigation of their variation pattern between the fractal dimension and its performance are valuable for establishing the relationship between pore structure and mechanical properties of ECC from microscopic aspects, as well as for the performance design of ECC. Therefore, in this study, the macroscopic properties and microstructure of ECC prepared from four different fine aggregates were investigated based on the previous studies, and the pore structure was tested by the mercury-pressure method. According to the thermodynamics-based pore structure fractal model previously proposed by authors [24], the relationship between the fractal dimension of ECCs with different fine aggregates and their micro–macro properties as well as pore structure characteristic parameters was explored to provide a reference idea for evaluating the performance of desert sand-based ECC by using the fractal dimension.

## 2. Materials and Methods

### 2.1. Materials and Mix Proportion

**Figure 1:**
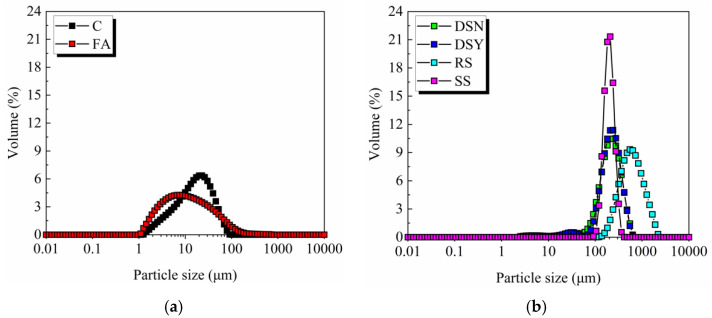
Particle size distributions of raw materials. (**a**) Powder material. (**b**) Different fine aggregates.

The raw materials used in this study included 42.5 ordinary Portland cement (C), fly ash (FA), four types of fine aggregates, tap water (W), high-efficiency water reducing agent (SP), redispersible latex powder (RLP) (supplied by Jinzhou Baoyi Building Materials Technology Co), and polyethylene (PE) fiber. Among them, four types of fine aggregates included untreated desert sand (DSN), washed desert sand (DSY), river sand (RS), and standard sand (SS). Figure 1 shows the particle size distribution of raw materials.

Table 1 lists the chemical composition of raw materials, measured by X-ray fluorescence (ARL PERFORM’X). Table 2 presents the basic physical properties of PE fibers, and Table 3 summarizes the specific mix proportions used in the test. Table 1 illustrates that the CaO content in FA is 13.37%, which is known as Class C high calcium fly ash by GB/T 1596 (Chinese Standard 2017) [25].

### 2.2. Test Methods

#### 2.2.1. Flowability

Flowability of fresh mortar was determined using GB/T 2419 (Chinese Standard 2005) [26] recommended jumping table test method. The fresh mortar was added into a high truncated cone die with the upper and lower diameter of 70 and 100 mm and wall thickness of 5 and 60 mm, respectively, and then pounded. Subsequently, the truncated cone die was gently lifted vertically upward, with a frequency of once per second moving 25 times, and then the diffusion diameter of the paste was quickly measured. The average value in the two vertical direction was measured as the test results of the flow. The reported results were calculated as the average of three independent tests to obtain the reliable results.

#### 2.2.2. Specimen Molding

After the completion of the flowability tests, the fresh ECC mixtures were cast into the corresponding molds for compression and uniaxial tension tests. Specimens were placed in a natural environment with a temperature of 18 ± 2 °C and relative humidity of 65 ± 5%. After 24 h, they were demolded and numbered. Next, the samples were placed in the standard curing box (temperature = 20 ± 2 °C and relative humidity = 95 ± 5%) for 28 d.

#### 2.2.3. Macroscopic Mechanical Properties

**Figure 2:**
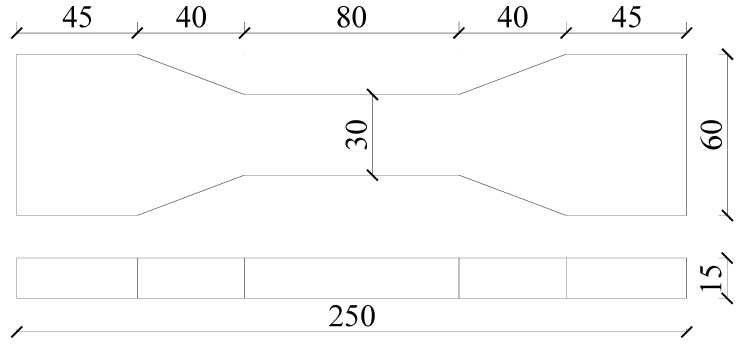
Dimensions of the dog bone specimen for the uniaxial tension tests.

The compression test was carried out on three cube-shaped specimens each with a dimension of 70.7 × 70.7 × 70.7 mm^3^ according to JGJ/T 70-2009 [27]. The uniaxial tension test was carried out on three dog bone-shape specimens, and their dimensions are shown in Figure 2. By referring to JC/T 2461-2018 [28], tensile deformation was measured using an extensometer with a loading rate of 0.5 mm·min^–1^. After the tensile test was completed, the average crack spacing and average crack width were obtained, while the number of cracks was calculated.

#### 2.2.4. Microstructure Characterization

After the corresponding tensile test, the central part of the specimens was taken and soaked in anhydrous ethanol to terminate the hydration reaction of the material, and then dried in an oven at 60 °C for 48 h for X-ray diffraction (XRD) and scanning electron microscopy/energy-dispersive spectroscopy (SEM/EDS) testing. SEM (TESCAN MIRA LMS) was used to characterize the microscopic morphology of ECC specimens prepared with different aggregates. Before the test, the sample was made into a square block of size no more than 5 mm. Furthermore, the elements in some areas of the sample were analyzed using an energy spectrometer attached to the electron microscope. The resolution of the instrument was 1.2 nm-30 keV, 3.5 nm-1 keV, the acceleration voltage was 200 eV–30 keV, and the magnification was 2–1,000,000 times. XRD (BrukerAXS D8) study was used for qualitative analysis of the phase compositions in ECC powder samples with different fine aggregates. The samples were taken from the middle part of the tensile failure sample, which were crushed and sampled after hydration and drying, and then ground in a mortar and sieved with a sieve having a mesh size of 200 μm. The sieve residue powder was the representative sample, and the sample dosage was generally more than 0.5 g. The instrument target was Cu target, the tube voltage was 40 kV, the current was 30 mA, the scanning rate was 5°·min^−1^, and the scanning angle was 5–90°.

The pore structure of cubic samples with the size of no more than 15 mm from the middle of the tensile failure sample was tested using a pore size analyzer (mercury intrusion porosimetry, MIP, AutoPore 9500). The pressure range of the instrument was 0.5–33000 psi, and the pore size range was 350 μm–5 nm. Thermodynamic models are usually used for the study of fractal dimension [24], and the calculation of the fractal dimension *D* is as follows [29].

(1) $$W_{n}=\sum_{i}^{n}\bar{p_{i}}\Delta V_{i}$$

(2) $$Q_{n}=\frac{V_{n}^{1/3}}{d_{n}}$$

(3) $$\mathrm{lg}\left(\frac{W_{n}}{d_{n}^{2}}\right)=D\mathrm{lg}Q_{n}+\mathrm{lg}C$$

where $\bar{p_{i}}$ is the average pressure of the ith mercury feeding operation, Pa, $\Delta V_{i}$ is the amount of mercury fed in the ith feeding operation, m^3^, *n* is the number of intervals applied in the mercury feeding operation, *d_n_* is the pore diameter corresponding to the nth mercury feeding, m, *V_n_* is the cumulative amount of mercury fed in pressure intervals 1 to *n*, m^3^; *C* is a constant; *W_n_* is the accumulated surface energy in the mercury intrusion process up to the nth stage, *Q_n_* is the function of pore radius rn and pore volume *V_n_* at the nth stage of mercury intrusion process, and *D* is the fractal dimension calculated based on thermodynamic relations. The corresponding groups of $W_{n}/d_{n}^{2}$ and *Q_n_* were obtained from the experimental mercury compression data, and then the logarithm of these two was plotted for linear regression. The slope of the obtained curve was the fractal dimension *D*.

## 3. Results and Discussion

### 3.1. Flowability

**Figure 3:**
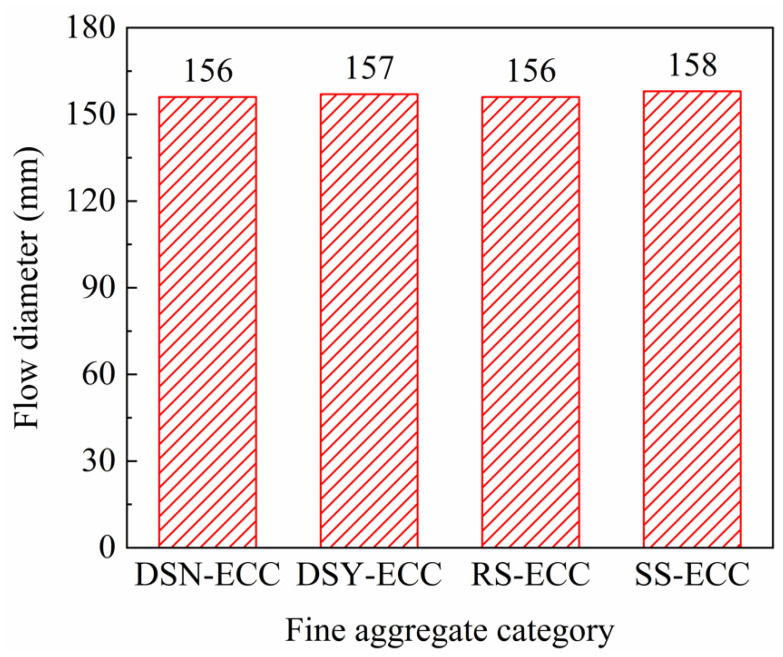
Flow diameter of different fine aggregate fresh pastes.

The working performance of ECC was measured by testing the maximum flow diameter of fresh mortars. Figure 3 illustrates that the flow diameter of the ECC mortars prepared from different fine aggregates is between 156 and 158 mm, and the flowability of the four mixtures is not much different. According to the literature [30], the influence of aggregates on the working properties of materials is mainly determined by their particle size distribution and structural characteristics. The better roundness of desert sand particles helps to reduce the friction between particles and improve the workability. However, Figure 1b presents that the particles in desert sand are finer compared to river sand, which can lead to a larger specific surface area and increased water demand. Therefore, the flowability of desert sand-based ECC is comparable with that of ECC prepared from river sand or standard sand under the mutual influence.

### 3.2. Compressive Strength

**Figure 4:**
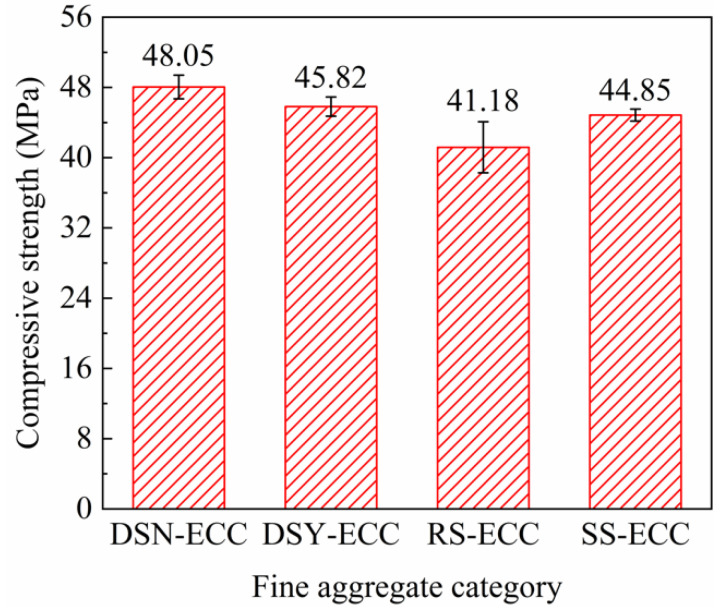
Relationship between fine aggregate category and compressive strength of ECC.

Figure 4 summarizes the compressive strength of ECC corresponding to different aggregates. Figure 4 illustrates that the order of compressive strength of ECC is: DSN-ECC > DSY-ECC > SS-ECC > RS-ECC, and the strength of DSN-ECC could reach 1.16 times that of RS-ECC. The results indicate that the ultra-fine particles with a particle size of less than 175 μm in desert sand exhibited heterogeneous nucleation and pozzolan effects. Particle size led to the reduction in the distance between nucleation sites and cement particles, promoted the hydration reaction of cement, and resulted in the generation of more hydration products, which not only optimized the matrix structure, but also enhanced the compressive strength of the matrix [31,32]. Furthermore, smaller desert sand particles and thinner slurry thickness can improve the structure and strength of the interfacial transition zone by inhibiting ion migration in the interfacial transition zone [33]. Moreover, the CaO/SiO_2_ ratio (calculated from Table 1: 0.166) is larger for desert sand compared to that for river sand and standard sand, which also exhibits a positive effect on the development of compressive strength [34].

### 3.3. Ultimate Tensile Performance

**Figure 5:**
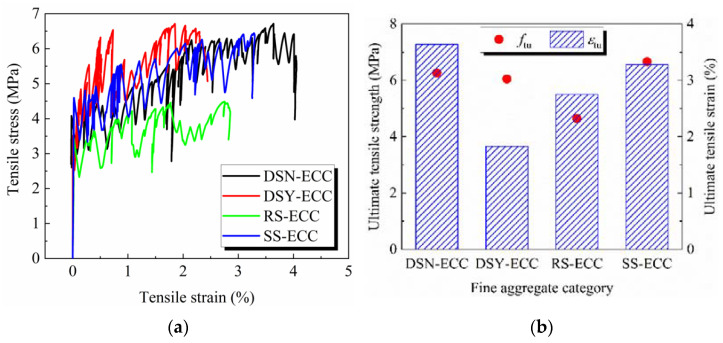
The fine aggregate category change group for (**a**) tensile stress–strain curves of ECC; (**b**) the relationship ultimate tensile strength and ultimate tensile strain of ECC.

The tensile stress–strain curves of ECC prepared from different fine aggregates are shown in Figure 5a. Figure 5b shows the changes of ultimate tensile strength and ultimate tensile strain of ECC under different fine aggregates, and the main characteristics are summarized in Table 4. The table illustrates that *f*_fc_, *f*_tu_, and *ɛ*_tu_ are the initial cracking strength, ultimate tensile strength, and ultimate tensile strain of the specimens, respectively.

Figure 5a intuitively shows that the ECC prepared from different fine aggregates show good ductility, and the stress continues to increase with the increase of strain until failure. Among them, DSN-ECC exhibits the best overall tensile performance, and RS-ECC shows the worst tensile performance.

Figure 5b demonstrates that the ultimate tensile strength of SS-ECC is the largest at 6.66 MPa, and the strength order of the remaining three ECCs is DSN-ECC > DSY-ECC > RS-ECC. The peak intensity of DSN-ECC is 6.26 MPa, reaching 94% that of SS-ECC. The order of ultimate tensile strain is as follows: DSN-ECC > SS-ECC > RS-ECC > DSY-ECC. DSN-ECC shows the largest ultimate tensile strain, reaching 3.638%, which is 1.99 times that of DSY-ECC. Desert sand particles are small and well rounded, which is advantageous for fiber dispersion and fiber bridging. On the other hand, the desert sand underwent some changes in its composition after washing with water (see Table 1), among which the reduction in Ca content may be the reason for the decrease in its tensile properties. Standard sand has a rougher appearance than desert sand, and the uneven surface leads to the increase in the friction with the fibers, which is beneficial for strength development. However, it weakens the fiber bridging, and therefore, leads to weakening of ultimate tensile strain. The large particle size of river sand can adversely affect the dispersion of fibers, resulting in poorer tensile properties [35].

### 3.4. SEM Analysis

**Figure 6:**
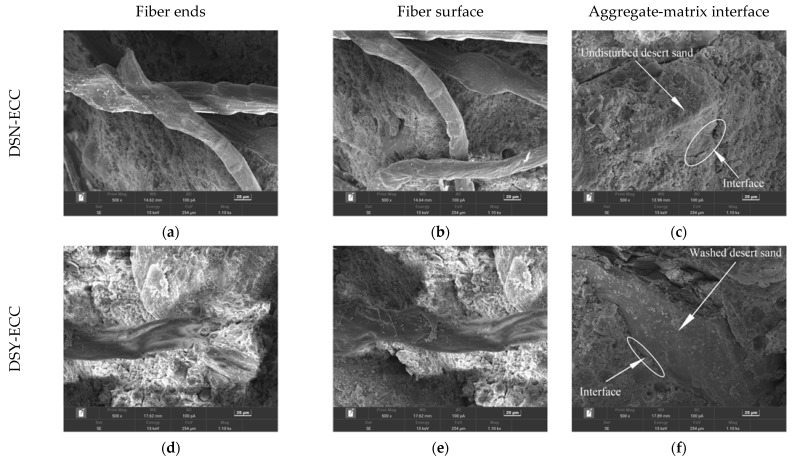

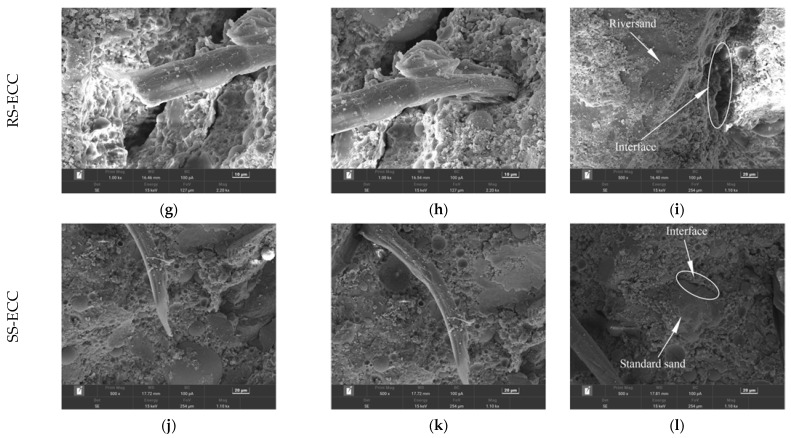
ECC microstructure of different fine aggregates.

SEM was used to observe the tensile damage sections of ECC specimens prepared with different fine aggregates, and the microscopic morphology of the transition zone at the interface between the aggregate and the matrix and the fibers was mainly observed. The corresponding results are shown in Figure 6, wherein Figure 6a–c show images for DSN-ECC, Figure 6d–f for DSY-ECC, Figure 6g–i for RS-ECC, and Figure 6j–l show images for SS-ECC.

Figure 6b reveals the presence of more intact fiber ends and obvious pull-out marks on the surface of the DSN-ECC samples, which indicates that the fibers were slowly sliding in the matrix rather than being pulled off directly from the matrix, i.e., the PE fibers in DSN-ECC produced good crack bridging capacity. Furthermore, Figure 6c shows that the transition zone at the interface between the matrix and the aggregate has a denser structure, resulting in excellent mechanical properties of the matrix, which may be the reason for the optimal tensile properties of DSN-ECC.

Figure 6c exhibits that the DSN-ECC matrix is well compacted and the desert sand is tightly wrapped by the matrix material. At the same time, combined with the EDS elemental analysis presented in Table 5, both Ca/Si and Al/Si of the products generated at the interface bond between the desert sand and the matrix are higher in DSN-ECC than in DSY-ECC, implying that the untreated desert sand may have undergone a volcanic ash reaction, while promoting the hydration of the surrounding cement and producing more dense gels [36,37]. As a result, the desert sand is more tightly bound to the matrix, which will facilitate the development of strength in DSN-ECC. Figure 6d,e presents that the fibers in DSY-ECC are most severely damaged with clear filamentary damage, which shows a worse effect on the tensile properties of the specimens. Figure 6g,h shows that the fiber ends of the river sand ECC specimens are relatively intact and have few surface scratches, which indicates that the fibers do not play a good bridging role. Furthermore, Figure 6i exhibits that some gaps appear between the river sand and the matrix, which indicates that the weak zone at the interface between the river sand and the matrix may be damaged first under mechanical action, affecting the mechanical property development. Figure 6j,k reveals that the fiber ends of SS-ECC were fractured and a large number of obvious scratches were produced on the fiber surface, indicating that the fibers also underwent bridging action. However, fibers were pulled off due to the tight bond with the fiber matrix, which is detrimental to the ductility development of the specimen, but may enhance the tensile strength.

### 3.5. X-Ray Diffraction Analysis

**Figure 7:**
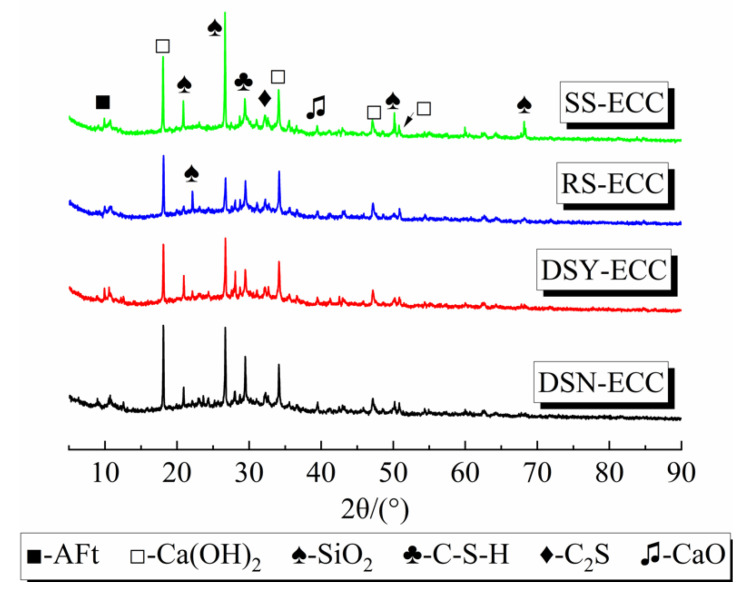
XRD patterns of hydrate of ECC under different fine aggregates.

The composition of the matrix phases of the four ECC is shown in Figure 7. The figures show that the main phases in the four types of ECCs are AFt, Ca(OH)_2_, silica (SiO_2_), C–S–H gel, and calcium oxide (CaO), and a small amount of dicalcium silicate (C_2_S). Moreover, AFt, Ca(OH)_2_, and C–S–H gel are the main hydration products of ECC. Compared with the other three sands, the peak intensities of C–S–H gels (29.4°) in the XRD spectrum of DSN-ECC are the largest, which indicates that more C–S–H gels are produced in desert sand-based ECC. This may be attributed to the volcanic ash effect of some active fines in the desert sand, which promotes the cement hydration [36]. In turn, the matrix structure was optimized, which led to an effective improvement of all aspects of DSN-ECC performance. In contrast, DSY-ECC produced less C–S–H gel than DSN-ECC, probably because some active desert sand particles were washed away during the washing process. This attenuated the hydration process of DSY-ECC and resulted in poorer mechanical properties.

### 3.6. Pore Structure Parameters

**Figure 8:**
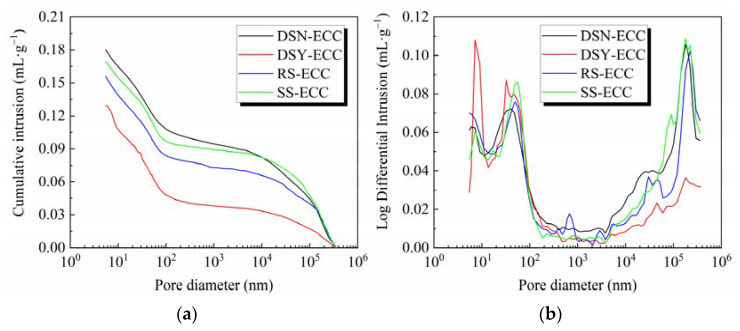
Pore structure parameters of different fine aggregates for (**a**) curve of cumulative mercury intake; (**b**) differential curve of pore diameter distribution.

Figure 8 presents the cumulative mercury intake curve and pore size distribution differential curve of ECC under four fine aggregates, and Table 5 lists the specific pore structure characteristic parameters. Among them, the critical pore size is defined as the pore size at the beginning of the steeply descending section of the curve in the cumulative mercury intake-pore size curve. Critical pore size is defined as the maximum pore level of each pore that can connect the larger pores, which effectively reflects the pore connectivity. The most probable pore size is the peak corresponding to the pore size in the pore size differential curve, which represents the most concentrated range of pore sizes in the matrix and is closely related to the pore size distribution pattern [38].

Figure 8 and Table 6 present that among the ECCs prepared with four different fine aggregates, DSY-ECC showed the smallest total pore volume, porosity, and average pore size; however, DSN-ECC exhibited the largest total pore volume, porosity, and average pore size. In other words, compared to DSN-ECC, the porosity and average pore size of the matrix got reduced more significantly after the washing away of the desert sand from the composite. The void structure is optimal even when compared to SS-ECC and RS-ECC, which indicates that the water washing of desert sand is advantageous for producing a matrix with excellent pore structure. This may be attributed to the reduction of the very fine particles in the desert sand after water washing, thus it has a greater effect on its pore structure. Moreover, the critical pore sizes of both DSN-ECC and DSY-ECC are the largest, indicating that the inter-pore connectivity of ECC prepared from desert sand is strong, which also implies that very fine particles exhibit little effect on the inter-pore connectivity of ECC materials. The small critical pore sizes of RS-ECC and SS-ECC indicate poor inter-pore connectivity, which may be attributed to the effect of their own morphology. For the most pore size, DSY-ECC is the smallest, which indicates that the most probable pore size in its matrix is small, while RS-ECC shows the largest pore size, which may be related to its particle gradation.

**Figure 9:**
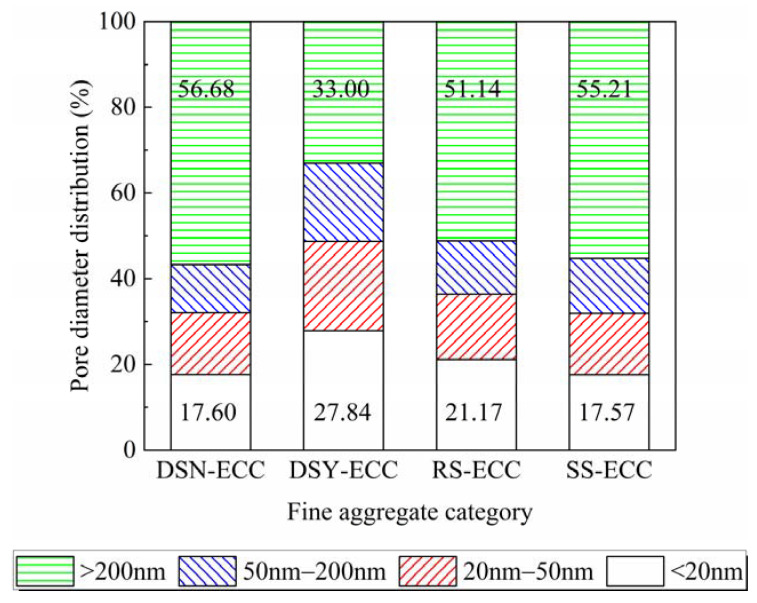
Pore diameter distribution of ECC under different fine aggregates.

Figure 9 presents a comparison chart of the content of different pore sizes in the total pore size of different fine aggregates. According to the pore size, the pores can be divided into harmless (<20 nm), less harmful (20–50 nm), harmful (50—200 nm), and multi-harmful (>200 nm) [39]. The figure illustrates that DSN-ECC and SS-ECC have low harmless pore content and DSN-ECC shows high harmless pore content. However, DSN-ECC and SS-ECC exhibit superior tensile strain resistance. It indicates that the more the content of harmless pores in ECC, the detrimental they are to the development of tensile strain resistance of the specimens.

### 3.7. Fractal Dimension

**Figure 10:**
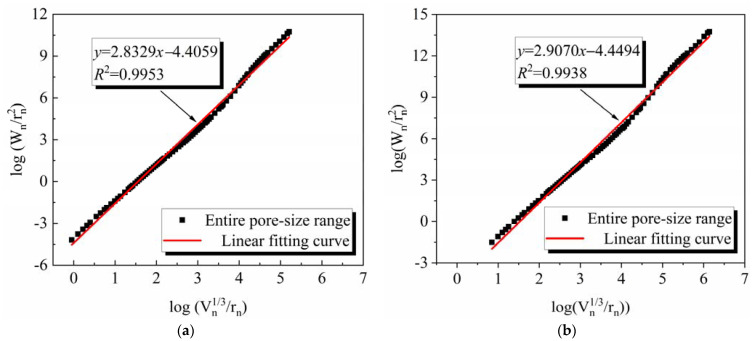

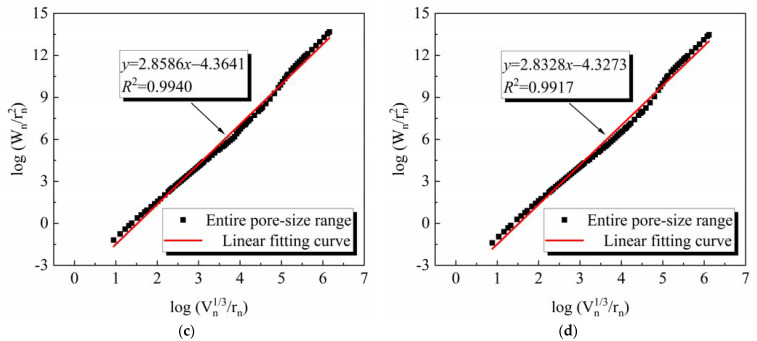
Fractal properties of pore structure in ECC with different fine aggregates. (**a**) DSN-ECC. (**b**) DSY-ECC. (**c**) RS-ECC. (**d**) SS-ECC.

The fractal dimension D of different ECCs was calculated based on the MIP data, and the results are presented in Table 7, and the specific pore structure fractal characteristics and their fitting results are shown in Figure 10.

Based on the principle of fractal theory, when the fractal dimension D value is equal to 2, it indicates that the object under test is a smooth plane, while D values close to 3 imply that the morphology and spatial distribution of the pores are very complex [40,41]. Therefore, D values between 2 and 3 [17,20,42] for the pore structure are meaningful. Table 7 presents that the D values of all ECC specimens vary between 2.8 and 2.9, which indicates the pore structure of ECC prepared with different fine aggregates shows a clear fractal character. Meanwhile, the fractal dimension provides a good characterization of the pore structure of ECCs with different fine aggregates. Table 7 presents that for ECC prepared with different aggregates, the D values are as follows: DSY-ECC > RS-ECC > DSN-ECC > SS-ECC and the DSY-ECC has the most complex pore structure. The above results indicate that the complexity of the pore structure of ECC prepared from different fine aggregates is different, which may be related to the particle sizes and chemical composition of different fine aggregates.

### 3.8. Analysis of Relationship between Fractal Dimension and Properties

#### 3.8.1. Relationship between Fractal Dimension and Compressive Strength

**Figure 11:**
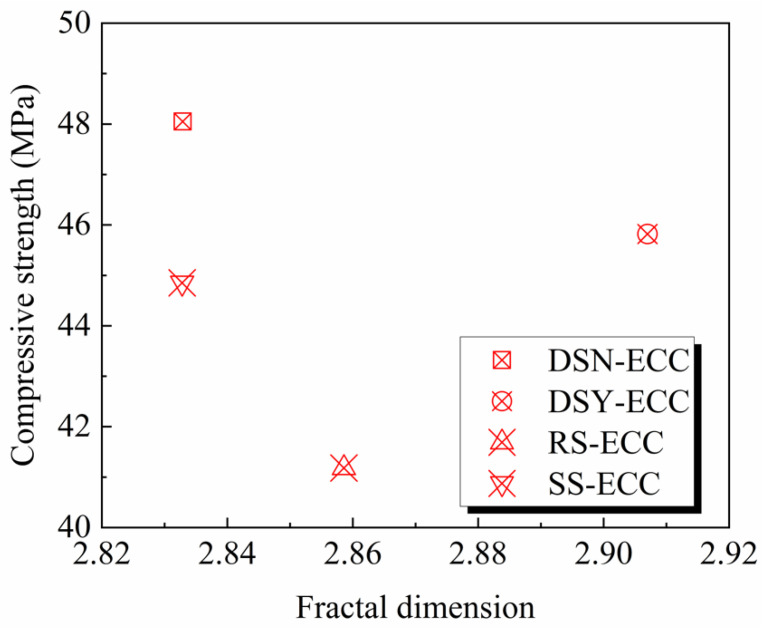
The relationship between fractal dimension and compressive strength.

Figure 11 shows the relationship between the fractal dimension and compressive strength of ECC. The figure illustrates the absence of any strong correlation between the compressive strength of ECC and the fractal dimension, which is consistent with the research results presented in a literature study [23], but different from the research results of other related studies [43,44,45], which may be related to the composite characteristics of ECC itself. It is not difficult to find that the water-washed desert sand leads to a more complex pore structure and also a decrease in compressive strength compared to DSN-ECC, which indicates that the water-washing process is detrimental to the development of mechanical properties of desert sand-based ECC.

#### 3.8.2. Relationship between Fractal Dimension and Ultimate Tensile Performance

**Figure 12:**
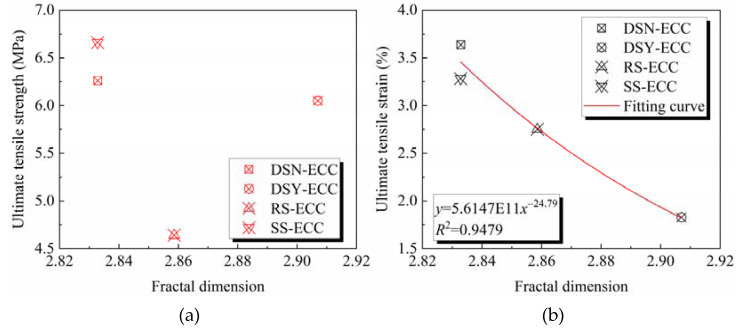
Relationship between fractal dimension and (**a**) ultimate tensile strength; (**b**) ultimate tensile strain.

Figure 12 show the relationship between the fractal dimension and the tensile properties of ECC. Figure 12a demonstrates that the fractal dimension of ECC with different aggregates is weakly correlated with the ultimate tensile strength, which is consistent with the findings on compressive strength. Compared to desert sand and standard sand, river sand shows a large particle size (see Table 1), and this may affect issues such as fiber dispersion and pore structure in ECC, resulting in large differences in mechanical properties. However, comparative analysis of the desert sand-based ECC and standard sand-based ECC with similar particle size indicates that the fractal dimension is negatively correlated with the compressive strength and tensile strength. That is, the larger the fractal dimension, the more unfavorable it is to the strength development. Figure 11 illustrates that with the increase of the fractal dimension, the ultimate tensile strain gradually decreases. Fitting of the relationship shows that the fractal dimension of ECC has a power function relationship with the ultimate tensile strain, and R^2^ is 0.9479, which is in good agreement. It indicates that the fractal dimension can be used to characterize and calculate the ultimate tensile strain of ECC.

#### 3.8.3. Relationship between Fractal Dimension and Pore Structure Parameters

**Figure 13:**
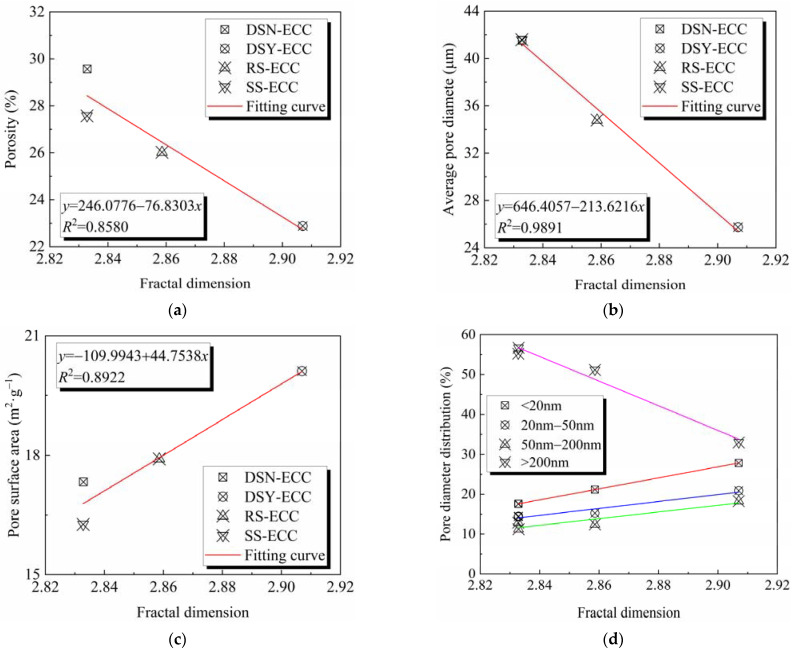
Relationship between fractal dimension and (**a**) porosity; (**b**) average pore diameter; (**c**) pore surface area; (**d**) pore diameter distribution.

Figure 13 displays the plot of fractal dimension versus pore structure parameters. The specific fitting result parameters of the fractal dimension and the pore diameter distribution are presented in Table 8.

Figure 13a,b exhibit that with the increase in the fractal dimension, the porosity and average pore size gradually decrease, and this is different from the results presented in the literature report [9], which may be the effect of the existence of aggregate category. The fitting of the results shows that the fractal dimension exhibits a linear relationship with the pore surface area and the average pore diameter, and R^2^ is 0.8580 and 0.9891, respectively, which is within the acceptable range. Figure 13c shows that with the increase in the fractal dimension, the pore surface area gradually increases, which is consistent with the results reported in the literature [9]. The fitting results show that the fractal dimension is linearly related to the pore surface area, and R^2^ is 0.8922, which is within the acceptable range. Figure 13d and Table 8 present that with the increase in the fractal dimension, the pore size content changes inconsistently, among which the pore size content in the range of <20 nm, 20–50 nm, and 50–200 nm gradually increases with the increase of fractal dimension; however, the pore size content of >200 nm gradually decreases. The relationships were fitted and found to be linear, and the agreement was good. Thus, clearly, the fractal dimension can be used to characterize and calculate the pore structure parameters of ECC.

## 4. Conclusions

In this study, the flowability, mechanical properties, and microstructure of polyethylene fiber-engineered cementitious composites (PE-ECC) with different fine aggregates were investigated, and the pore structure of PE-ECC was analyzed using fractal dimension. Based on the results, the following conclusions can be drawn.

(1) For ECC flowability, aggregate type exhibits little effect on it. Compared with river sand, standard sand, and washed desert sand, the comprehensive performance of untreated desert sand ECC is superior, and desert sand as fine aggregate for PE-ECC production offers certain advantages.
(2) The presence of certain active powder particles in the desert sand synergistically promotes the hydration reaction of cement and generates more C–S–H gel, which closely combines the desert sand with the matrix and leads to effective improvement in the mechanical properties of the matrix, such as compressive strength and tensile strength. The mechanical properties of the desert sand ECC get reduced after water washing, which may be the reason for the washing away of some of the active micronized powder in the desert sand.
(3) The fractal dimension of the ECC prepared from the four sands varies in size, but all are within the range of 2.8–2.9, indicating that they have obvious fractal characteristics. Moreover, the correlation fit coefficients are all greater than 0.99, indicating that the fractal model assumed in this study shows good applicability.
(4) The fractal dimension is not significantly correlated with the compressive strength and ultimate tensile strength of ECC, but excluding river sand, the fractal dimension of the other three sands is negatively correlated with the tensile strength and compressive strength. The ultimate tensile strain decreases with the increase of the fractal dimension, showing a good correlation.
(5) The fractal dimension exhibits good correlation with the pore structure parameters, wherein porosity and average pore size are negatively correlated with fractal dimension, and pore surface area is positively correlated with fractal dimension. The higher the content of large pores (>200 nm), the smaller the fractal dimension. In contrast, the higher the number of pores <200 nm, the larger the fractal dimension.

## Figures and Tables

**Table 1:** Chemical compositions of raw materials.

Compositions (wt. %)	CaO	SiO_2_	Al_2_O_3_	Fe_2_O_3_	MgO	SO_3_	K_2_ O	Na_2_O	Others
C	65.26	18.69	3.95	4.32	1.52	3.72	0.62	0.83	1.09
FA	13.37	45.68	16.72	10.42	4.37	1.73	2.10	3.18	2.43
DSN	9.52	57.18	12.84	7.46	2.77	2.38	2.61	3.28	1.96
DSY	8.03	61.97	12.85	6.30	2.10	1.09	2.55	3.61	1.50
RS	2.85	69.97	12.68	4.80	1.63	0.13	3.58	3.32	1.04
SS	1.01	90.98	3.42	0.56	0.20	0.63	1.96	0.26	0.98

**Table 2:** Basic physical properties of PE fibers.

Length/mm	Diameter/μm	Tensile strength/MPa	Tensile modulus/GPa	Elongation at break/%	Density/(g·cm^−3^)
12	24	3000	110	2–3	0.98

**Table 3:** Mix proportion (by weight ratio) used in the test.

Mixtures	C	FA	Sand	W	SP	RLP	PE Fiber (vol%)
DSN-ECC	1.0	1.5	1.55	0.34	0.004	0.004	1.5
DSY-ECC							
RS-ECC							
SS-ECC							

**Table 4:** Uniaxial tensile properties of ECC in different fine aggregates.

Mixtures	*f*_fc_/MPa	*f*_tu_/MPa	*ɛ*_tu_/%
DSN-ECC	2.74	6.26	3.638
DSY-ECC	2.63	6.05	1.827
RS-ECC	2.49	4.64	2.752
SS-ECC	3.16	6.66	3.282

**Table 5:** Elemental composition and content of different fine aggregates at the junction with the matrix (%).

Mixtures	O	Na	Mg	Al	Si	K	Ca	Fe	Ca/Si	Al/Si
DSN-ECC	38.51	1.08	3.24	8.22	14.66	0.69	25.24	8.36	1.722	0.561
DSY-ECC	36.34	0.21	2.02	2.64	30.76	0.45	23.57	4.01	0.766	0.086
RS-ECC	49.07	0.67	0.51	2.33	31.14	0.28	14.39	1.61	0.462	0.075
SS-ECC	39.24	0.00	0.15	0.54	22.05	0.04	37.38	0.60	1.695	0.024

**Table 6:** Characteristic parameters of pore structure under different fine aggregate.

Mixtures	Pore Volume/(mL-g^−1^)	Porosity/%	Average Pore size/nm	Critical Pore size/nm	Most Probable Pore Size/μm
DSN-ECC	0.1800	29.57	41.53	120.76	178.971
DSY-ECC	0.1294	22.88	25.74	120.76	0.007
RS-ECC	0.1558	26.02	34.79	95.44	223.637
SS-ECC	0.1691	27.57	41.59	95.32	179.206

**Table 7:** The D values of ECC with different fine aggregates.

Mixtures	D	R^2^
DSN-ECC	2.8329	0.9953
DSY-ECC	2.9070	0.9938
RS-ECC	2.8586	0.9940
SS-ECC	2.8328	0.9917

**Table 8:** Fractal dimension and pore size distribution fitting result parameters.

	Parameters	Formula	a	b	R^2^
Pore Volume Fractions					
<20 nm		y=a+b∗x	−374.2093	138.3060	0.9999
20 nm–50 nm			−232.2389	86.9600	0.9234
50 nm–200 nm			−225.2806	83.6215	0.8101
>200 nm			931.3645	−308.7512	0.9639

## Data Availability

Not applicable.
